# Inclisiran in Patients with CKD: *Post Hoc* Pooled Analysis of Three Phase 3 Trials

**DOI:** 10.1681/ASN.0000001006

**Published:** 2026-01-28

**Authors:** Ulf Landmesser, Kausik K. Ray, Frederick J. Raal, Lorena Garcia Conde, Jackie Han, Wolfgang Koenig, Lawrence A. Leiter, Gregory G. Schwartz, R. Scott Wright

**Affiliations:** 1 Department of Cardiology, Angiology and Intensive Care Medicine, Deutsches Herzzentrum der Charité, Charité-Universitätsmedizin Berlin, Berlin Institute of Health, DZHK, Partner Site Berlin, Friede Springer Cardiovascular Prevention Center at Charité, Berlin, Germany; 2 Department of Primary Care and Public Health, Imperial Centre for Cardiovascular Disease Prevention, Imperial College, London, United Kingdom; 3 Department of Medicine, Faculty of Health Sciences, University of the Witwatersrand, Johannesburg, South Africa; 4 Novartis Pharma AG, Basel, Switzerland; 5 Novartis Pharmaceuticals Corporation, East Hanover, New Jersey; 6 German Heart Centre, School of Medicine and Health, TUM University Hospital, Technical University of Munich, Munich, Germany; 7 DZHK (German Centre for Cardiovascular Research), Partner Site Munich Heart Alliance, Munich, Germany; 8 Institute of Epidemiology and Medical Biometry, University of Ulm, Ulm, Germany; 9 Li Ka Shing Knowledge Institute, St. Michael's Hospital, University of Toronto, Toronto, Ontario, Canada; 10 Division of Cardiology, University of Colorado School of Medicine, Aurora, Colorado; 11 Division of Preventive Cardiology, Department of Cardiology, Mayo Clinic, Rochester, Minnesota

**Keywords:** atherosclerosis, atherosclerotic cardiovascular disease, CKD, eGFR, inclisiran, lipid therapy, low-density lipoprotein cholesterol, PCSK9 inhibitor

## Abstract

**Key Points:**

- Inclisiran for LDL cholesterol reduction was analyzed *post hoc* in patients with CKD.
- Mean percentage LDL cholesterol reduction from baseline was around 50% regardless of eGFR in phase 3 trials.
- Inclisiran showed sustained and effective LDL cholesterol–lowering in patients across various levels of eGFR values, without new safety findings.

**Background:**

Lowering LDL cholesterol reduces the risk of atherosclerotic cardiovascular disease in patients with CKD. The efficacy and safety of inclisiran versus placebo in patients without and with CKD were investigated in a *post hoc* pooled analysis of three phase 3 trials (ORION-9, ORION-10, and ORION-11).

**Methods:**

Patients with heterozygous familial hypercholesterolemia, atherosclerotic cardiovascular disease or its risk equivalent, and elevated LDL cholesterol were randomized 1:1 to subcutaneous inclisiran or placebo on days 1 and 90 and every 6 months thereafter for 540 days. Patients were stratified based on baseline eGFR (by CKD Epidemiology Collaboration equation): ≥90, 60 to <90, 45 to <60, and 15 to <45 ml/min per 1.73 m^2^. Coprimary end points were percentage change in LDL cholesterol at day 510 and time-adjusted percentage change after day 90 and through day 540. Safety was also evaluated.

**Results:**

Of 3660 patients, 1610 (44%) had eGFR ≥90, 1608 (44%) 60 to <90, 300 (8%) 45 to <60, and 142 (4%) 15 to <45 ml/min per 1.73 m^2^. The mean (95% confidence interval) placebo-corrected percentage changes in LDL cholesterol from baseline at day 510 in patients with eGFR ≥90, 60 to <90, 45 to <60, and 15 to <45 ml/min per 1.73 m^2^ were −49.9% (−53.2 to −46.6), −51.2% (−54.4 to −48.0), −54.7% (−62.5 to −47.0), and −44.7% (−57.6 to −31.8), respectively (*P* < 0.001); the corresponding mean (95% confidence interval) time-adjusted placebo-corrected percentage changes in LDL cholesterol from baseline after day 90 through day 540 were −48.4% (−50.8 to −46.1), −51.8% (−54.2 to −49.4), −55.6% (−61.0 to −50.2), and −50.4% (−59.3 to −41.5; each *P* < 0.001). Significant decreases in total cholesterol, apolipoprotein B, non-HDL cholesterol, and lipoprotein(a) occurred in all eGFR groups. Inclisiran was well tolerated without new safety findings.

**Conclusions:**

Inclisiran demonstrated sustained and effective LDL cholesterol reduction in patients with or at risk of atherosclerotic cardiovascular disease, regardless of baseline eGFR as low as 15 ml/min per 1.73 m^2^, without new safety findings.

**Clinical Trial registry name and registration number:**

ClinicalTrials.gov, ORION-9 (NCT03397121), ORION-10 (NCT03399370), and ORION-11 (NCT03400800).

## Visual Abstract

**Figure:**
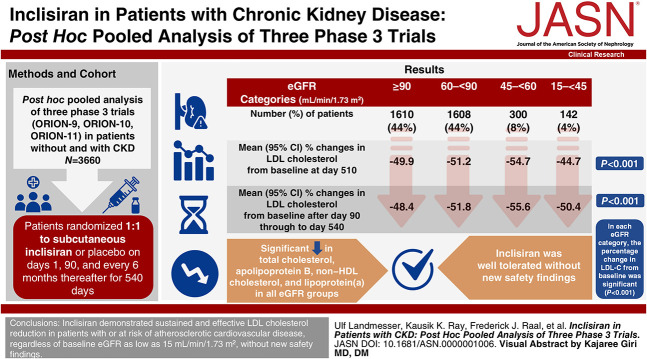

## Introduction

Patients with CKD experience a significantly higher risk of atherosclerotic cardiovascular disease events.^1,2^ More severe CKD, as reflected by lower eGFR, is associated with greater incidence of cardiovascular events and death.^3^ Furthermore, patients with prevalent atherosclerotic cardiovascular disease and CKD are at even higher risk of cardiovascular events.^4,5^ Present guidelines consistently classify patients with advanced CKD at high risk, but recommendations for LDL cholesterol treatment goals differ. The Kidney Disease Improving Global Outcomes (KDIGO) guideline recommends statin therapy for patients with CKD and atherosclerotic cardiovascular disease without specifying LDL cholesterol goals.^6^ The European Society of Cardiology (ESC) recommends LDL cholesterol goals of <1.4 mmol/L (<55 mg/dl) or <1.8 mmol/L (<70 mg/dl), plus an LDL cholesterol reduction of >50% from baseline, respectively, for patients considered at high or very high risk on the basis of eGFR <60 ml/min per 1.73 m^2^.^7^ Both the American College of Cardiology/American Heart Association and the ESC guidelines recommend statin therapy for patients with atherosclerotic cardiovascular disease and non–dialysis-dependent stages 3-5 CKD, regardless of baseline LDL cholesterol levels.^1,7^ However, some patients with CKD experience myopathy or statin intolerance,^8,9^ necessitating the use of nonstatin lipid therapies. Consequently, KDIGO, ESC, and American College of Cardiology/American Heart Association guidelines each indicate that nonstatin therapies, such as ezetimibe or proprotein convertase subtilisin/kexin type 9 (PCSK9) inhibitors, be considered for patients who do not achieve LDL cholesterol goals despite maximum-tolerated statin therapy.^1,6,7^ In fact, on a background of statin therapy, ezetimibe and anti-PCSK9 monoclonal antibodies have been shown to reduce the risk of cardiovascular events compared with placebo.^10–12^

Inclisiran, a first-in-class small interfering RNA agent, degrades PCSK9 mRNA in the liver to prevent PCSK9 production, resulting in increased LDL receptor concentration and reduced plasma LDL cholesterol levels.^13^ Pivotal phase 3 ORION-9 (NCT03397121), ORION-10 (NCT03399370), and ORION-11 (NCT03400800) trials demonstrated that twice-yearly (after initial and 3-month doses) inclisiran provides sustained and effective LDL cholesterol lowering and is well tolerated in patients with or at high risk of atherosclerotic cardiovascular disease.^14–16^ In addition, data from the phase 1 ORION-7 (*N*=31) and phase 2 ORION-1 (*N*=247) trials demonstrate that exposure to inclisiran sodium 300 mg reduced PCSK9 and LDL cholesterol levels consistently across CKD stages 1-4; patients requiring hemodialysis (stage 5) were excluded. Patients with more advanced CKD had higher initial maximum plasma concentrations of inclisiran after administration, but this heightened exposure did not affect LDL cholesterol reduction, and inclisiran was undetectable in plasma after 48 hours, suggesting that dose adjustments are not necessary across various stages of CKD.^17^ Studies ORION-7 and ORION-1 were able to provide follow-up to 210 days of evaluation. The *post hoc* pooled analysis of the ORION-9, ORION-10, and ORION-11 trials aimed to assess the efficacy and safety of inclisiran versus placebo in a large pool of patients, encompassing those without or with CKD, over an extended follow-up duration.

## Methods

### Study Design and Participants

The analysis cohort included participants from the pivotal phase 3 ORION-9, ORION-10, and ORION-11 double-blind, placebo-controlled, parallel-group trials.^14,15^ Qualifying patients had heterozygous familial hypercholesterolemia, atherosclerotic cardiovascular disease or its risk equivalent, and elevated LDL cholesterol (≥100 mg/dl for heterozygous familial hypercholesterolemia and atherosclerotic cardiovascular disease risk equivalent; ≥70 mg/dl for atherosclerotic cardiovascular disease) at screening despite maximally tolerated statins with or without additional oral lipid-lowering therapy. The maximum tolerated statin dose was defined as the maximum dose that can be taken on a regular basis without intolerable adverse events. For patients who are intolerant to statins, it was necessary to provide documented evidence of intolerance to the lowest approved dose of at least two different statins. Patients receiving anti-PCSK9 monoclonal antibodies were excluded.

Detailed inclusion and exclusion criteria have been published previously.^14,15^ In all three trials, participants were initially required to have a screening eGFR >30 ml/min per 1.73 m^2^ by eGFR using local clinical methodology. In the ORION-10 trial, the protocol was later amended to remove the eGFR criterion and to instead require no current or planned dialysis or kidney transplantation, therefore allowing inclusion of some patients with severe kidney impairment as measured at the local study site.

For this *post hoc* pooled analysis, all results were based on eGFR values estimated by the 2021 CKD Epidemiology Collaboration equation^18^ applied to creatinine measurements as provided by the central laboratory. Patients were classified into four categories based on their baseline eGFR. These categories align with KDIGO GFR categories: ≥90 ml/min per 1.73 m^2^ (KDIGO GFR category G1); 60 to <90 ml/min per 1.73 m^2^ (KDIGO GFR category G2); 45 to <60 ml/min per 1.73 m^2^ (KDIGO GFR category G3A); and 15 to <45 ml/min per 1.73 m^2^ (KDIGO GFR category G3B–G4). None of the patients had eGFR <15 ml/min per 1.73 m^2^, which aligns with KDIGO GFR category G5.

In the pivotal phase 3 trials, patients were randomized 1:1 to receive 300 mg inclisiran sodium (equivalent to 284 mg inclisiran) or placebo on days 1, 90, 270, and 450, with maximum-tolerated background oral lipid-lowering therapy. Follow-up visits for laboratory and safety assessments were conducted on days 30, 150, 330, and 510 and the end-of-trial visit on day 540 (Supplemental Figure 1). In all three trials, randomization was stratified according to background statin use and additionally according to country in the ORION-9 and ORION-11 trials. ORION-9 included patients from Canada, Europe, South Africa, and the United States; ORION-10 from the United States; and ORION-11 from Czech Republic, Germany, Hungary, The Netherlands, Poland, South Africa, Ukraine, and the United Kingdom. The study protocols were approved by the relevant Institutional Review Board/independent ethics committee, and all participants provided informed consent.

### Outcomes

The coprimary efficacy end points were the percentage change in LDL cholesterol from baseline at day 510 and time-adjusted percentage change in LDL cholesterol from baseline after day 90 up to day 540. The key secondary efficacy end points were the absolute change in LDL cholesterol from baseline at day 510 and time-adjusted absolute change in LDL cholesterol from baseline after day 90 up to day 540.

Other secondary end points included the percentage and absolute changes from baseline at day 510 in total cholesterol, non-HDL cholesterol, apolipoprotein B (ApoB), triglycerides, lipoprotein(a) (Lp(a) at day 540), and remnant cholesterol (this was derived *post hoc* for the analysis). Exploratory *post hoc* end points included the effects of inclisiran on eGFR outcomes such as the number of patients with a decline in eGFR by ≥30%, ≥40%, and ≥50% based on baseline eGFR status and the percentage change in eGFR over time. Safety end points included the proportion of patients with treatment-emergent adverse events (TEAEs), serious TEAEs, TEAEs leading to discontinuation, death, frequent TEAEs (reported in ≥5% of patients), and clinically significant laboratory values.

### Statistical Analysis

Baseline characteristics and efficacy were evaluated in the intent-to-treat (ITT) population comprising all randomized patients in the three trials. The percentage and absolute changes in LDL cholesterol from baseline to day 510 were analyzed using an analysis of covariance model with a multiple imputation washout model for missing data. The time-adjusted percentage and absolute changes in LDL cholesterol from baseline after day 90 up to day 540 was analyzed using a mixed-effects model for repeated measures with a control-based pattern mixture model for missing data imputation. The time-adjusted percentage (or absolute) change was defined as the average percentage (or absolute) change in LDL cholesterol from baseline after day 90 up to day 540 and was calculated from the respective mixed-effects model for repeated measures as a linear combination of the estimated means of the corresponding time points. Details of the multiple imputation methods and handling of missing data due to early discontinuation, including patients lost to follow-up, are available in Supplemental Appendix 1.

The percentage and absolute changes in other atherogenic lipids from baseline to day 510 were analyzed using a mixed-effects model for repeated measures without imputation. For Lp(a), median-based analysis at day 540 was provided from a quantile regression model without imputation of missing data. Models were applied separately within each eGFR categories. Tests for the interaction of treatment with eGFR categories were conducted for the key efficacy end points. Analyses by eGFR categories were not prespecified and, therefore, were not adjusted for multiplicity. Nominal *P* values and 95% confidence intervals (CIs) were considered as measures for the strength of the association between treatment arms and end points and not formal criteria to claim statistical significance. Safety was analyzed in the safety population comprising all patients who received ≥1 dose of the study drug.

## Results

### Demographics and Baseline Characteristics

In this pooled analysis, all 3660 randomized patients had available baseline eGFR levels and were classified into four groups: 1610 (44%) had eGFR ≥90, 1608 (44%) had 60 to <90, 300 (8%) had 45 to <60, and 142 (4%) had 15 to <45 ml/min per 1.73 m^2^. There were 14 patients (0.4%) with baseline eGFR <30 ml/min per 1.73 m^2^, all but one from ORION-10. This trial allowed patients with severe kidney impairment at screening and those with screening eGFR >30 ml/min per 1.73 m^2^ but presenting a lower value at baseline. No patient had baseline eGFR <15 ml/min per 1.73 m^2^, which would qualify for kidney failure. Owing to the limited number of patients with baseline eGFR 15 to <30 ml/min per 1.73 m^2^ and the imbalance between treatment groups (inclisiran *n*=10; placebo, *n*=4), robust results could not be generated using the statistical modeling techniques applied. Therefore, this subset was analyzed together with patients having baseline eGFR of 15 to <45 ml/min per 1.73 m^2^.

Baseline demographic and clinical characteristics were balanced between the inclisiran and placebo groups across all eGFR categories (Table 1). Overall, 3107 patients (85%) had atherosclerotic cardiovascular disease and 553 (15%) were atherosclerotic cardiovascular disease risk equivalent. Patients with lower rate of eGFR were older, more likely to be women, and more likely to have clinical atherosclerotic cardiovascular disease, heart failure, and hypertension and had a less intensive use of a lipid-lowering therapy. The mean LDL cholesterol at baseline was lower in patients with eGFR 15 to <45 ml/min per 1.73 m^2^ compared with patients with higher eGFR, likely due to the use of higher intensity lipid-lowering therapy in the former groups.

**Table 1:** Baseline demographic and clinical characteristics by baseline eGFR (intent-to-treat population)

Characteristic	eGFR ≥90 ml/min per 1.73 m^2^		eGFR 60 to <90 ml/min per 1.73 m^2^		eGFR 45 to <60 ml/min per 1.73 m^2^		eGFR 15 to <45 ml/min per 1.73 m^2^	
	Inclisiran (*n*=822)	Placebo (*n*=788)	Inclisiran (*n*=790)	Placebo (*n*=818)	Inclisiran (*n*=155)	Placebo (*n*=145)	Inclisiran (*n*=66)	Placebo (*n*=76)
Age, yr, mean±SD	60±10	59±10	67±8	67±9	69±8	71±7	72±7	69±7
Male, *n* (%)	557 (68)	548 (70)	529 (67)	564 (69)	96 (62)	93 (64)	44 (67)	39 (51)
eGFR, ml/min per 1.73 m^2^, median (Q1–Q3)	93 (85–103)	93 (86–102)	71 (65–76)	70 (64–76)	51 (46–53)	50 (46–53)	37 (32–39)	37 (34–40)
LDL cholesterol, mg/dl, mean±SD	116±47	115±45	108±40	108±44	111±53	105±35	103±40	104±35
**Atherosclerotic cardiovascular disease status, *n* (%)**								
Atherosclerotic cardiovascular disease	637 (77)	629 (80)	706 (89)	721 (88)	148 (95)	135 (93)	61 (92)	70 (92)
Atherosclerotic cardiovascular disease risk equivalent	185 (23)	159 (20)	84 (11)	97 (12)	7 (5)	10 (7)	5 (8)	6 (8)
**Cardiovascular risk factors, *n* (%)**								
Congestive heart failure	74 (9)	62 (8)	99 (13)	114 (14)	27 (17)	32 (22)	13 (20)	19 (25)
Hypertension	595 (72)	602 (76)	661 (84)	655 (80)	141 (91)	136 (94)	59 (89)	70 (92)
Diabetes mellitus	279 (34)	252 (32)	287 (36)	269 (33)	76 (49)	67 (46)	45 (68)	43 (57)
Hyperlipidemia	796 (97)	760 (96)	752 (95)	773 (94)	151 (97)	136 (94)	64 (97)	73 (96)
Familial hypercholesterolemia	205 (25)	193 (24)	113 (14)	133 (16)	14 (9)	14 (10)	8 (12)	12 (16)
**Lipid-lowering therapy, *n* (%)**								
Statins	764 (93)	732 (93)	727 (92)	749 (92)	136 (88)	126 (87)	59 (89)	68 (89)
High-intensity statin	626 (76)	629 (80)	578 (73)	570 (70)	104 (67)	95 (66)	48 (73)	51 (67)
Ezetimibe	146 (18)	136 (17)	82 (10)	111 (14)	20 (13)	12 (8)	3 (5)	11 (14)

Patients were classified into four categories based on their baseline eGFR. These categories align with Kidney Disease Improving Global Outcomes (KDIGO) GFR categories: ≥90 ml/min per 1.73 m^2^ (KDIGO GFR category G1); 60 to <90 ml/min per 1.73 m^2^ (KDIGO GFR category G2); 45 to <60 ml/min per 1.73 m^2^ (KDIGO GFR category G3A); and 15 to <45 ml/min per 1.73 m^2^ (KDIGO GFR category G3B–G4). None of the patients had GFR <15 ml/min per 1.73 m^2^, which aligns with KDIGO GFR category G5. Q1, lower quartile; Q3, upper quartile.

Overall, 237 (6%) patients discontinued the trials before the day 540 visit, including 46 (1%) patients considered lost to follow-up.

### Efficacy

#### Primary End Points

**Figure 1:**
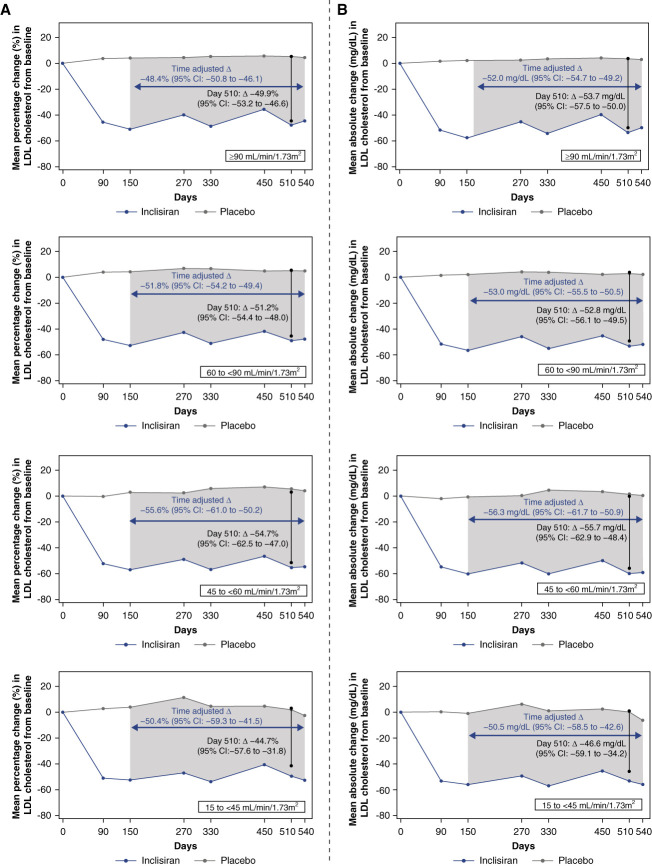
**Placebo-corrected LDL cholesterol** **change from baseline at day 510 and corresponding time-adjusted change from baseline after day 90 and up to day 540 by baseline eGFR (ITT population).** (A) Percentage change in LDL cholesterol and (B) absolute change in LDL cholesterol. Note: the percentage and absolute changes in LDL cholesterol from baseline to day 510 were analyzed using an ANCOVA model with a multiple imputation washout model for missing data. The time-adjusted percentage and absolute changes in LDL cholesterol from baseline after day 90 and up to day 540 was analyzed using a mixed-effects model for repeated measures with control-based pattern mixture model for missing data imputation. The displayed mean percentage (%) and absolute (mg/dl) changes over time are least squares mean values derived from mixed-effects model for repeated measures without imputation. All models were adjusted for baseline LDL cholesterol value and study and applied separately within each eGFR category. Treatment differences are presented for inclisiran − placebo. Patients were classified into four categories based on their baseline eGFR. These categories align with KDIGO GFR categories: ≥90 ml/min per 1.73 m^2^ (KDIGO GFR category G1); 60 to <90 ml/min per 1.73 m^2^ (KDIGO GFR category G2); 45 to <60 ml/min per 1.73 m^2^ (KDIGO GFR category G3A); and 15 to <45 ml/min per 1.73 m^2^ (KDIGO GFR category G3B–G4). None of the patients had GFR <15 ml/min per 1.73 m^2^, which aligns with KDIGO GFR category G5. Δ, treatment difference; ANCOVA, analysis of covariance; CI, confidence interval; ITT, intent-to-treat; KDIGO, Kidney Disease Improving Global Outcomes.

The mean percentage change in LDL cholesterol from baseline at day 510 and the corresponding time-adjusted percentage change from baseline after day 90 up to day 540 were significantly greater with inclisiran versus placebo (*P* < 0.001), irrespective of baseline eGFR category (Figure 1A). In patients with eGFR ≥90, 60 to <90, 45 to <60, and 15 to <45 ml/min per 1.73 m^2^, the mean (95% CI) placebo-corrected percentage changes in LDL cholesterol from baseline at day 510 were −49.9% (−53.2 to −46.6), −51.2% (−54.4 to −48.0), −54.7% (−62.5 to −47.0), and −44.7% (−57.6 to −31.8), respectively (*P* < 0.001, Figure 1A; treatment by eGFR subgroup interaction effect *P* = 0.42; Supplemental Table 1). The corresponding mean (95% CI) time-adjusted placebo-corrected percentage changes in LDL cholesterol from baseline after day 90 up to day 540 were −48.4% (−50.8 to −46.1), −51.8% (−54.2 to −49.4), −55.6% (−61.0 to −50.2), and −50.4% (−59.3 to −41.5) (each *P* < 0.001, Figure 1A; treatment by eGFR subgroup interaction effect *P* = 0.11; Supplemental Table 1).

#### Key Secondary End Points

The mean absolute change in LDL cholesterol from baseline at day 510 and the corresponding time-adjusted absolute change from baseline after day 90 up to day 540 were significantly greater with inclisiran versus placebo (*P* < 0.001), irrespective of baseline eGFR category (Figure 1B). In patients with eGFR ≥90, 60 to <90, 45 to <60, and 15 to <45 ml/min per 1.73 m^2^, the mean (95% CI) placebo-corrected absolute changes in LDL cholesterol (mg/dl) from baseline at day 510 were −53.7 (−57.5 to −50.0), −52.8 (−56.1 to −49.5), −55.7 (−62.9 to −48.4), and−46.6 (−59.1 to −34.2), respectively (each *P* < 0.001, Figure 1B; treatment by eGFR subgroup interaction effect *P* = 0.55; Supplemental Table 1). The corresponding mean (95% CI) time-adjusted placebo-corrected absolute changes in LDL cholesterol (mg/dl) from baseline after day 90 and up to day 540 was −52.0 (−54.7 to −49.2), −53.0 (−55.5 to −50.5), −56.3 (−61.7 to −50.9), and −50.5 (−58.5 to −42.6) mg/dl, respectively (each *P* < 0.001, Figure 1B; treatment by eGFR subgroup interaction effect *P* = 0.71; Supplemental Table 1).

#### Other Secondary End Points

The placebo-corrected percentage changes in total cholesterol, ApoB, non–HDL cholesterol, triglycerides, Lp(a), and remnant cholesterol from baseline to day 510 (day 540 for Lp[a]) were significantly greater with inclisiran than with placebo, regardless of eGFR category. The placebo-corrected absolute changes correlated with their respective percentage changes (Table 2). The interactions of treatment and eGFR subgroups were not statistically significant for most atherogenic lipids with the exception of triglycerides and remnant cholesterol (Supplemental Table 1).

**Table 2:** Placebo-corrected percentage and absolute changes in other atherogenic lipids at day 510

Parameters	Percentage Change, %, LS Mean (95% CI)^a^				Absolute Change, mg/dl, LS Mean (95% CI)^a^			
	eGFR ≥90 ml/min per 1.73 m^2^	eGFR 60 to <90 ml/min per 1.73 m^2^	eGFR 45 to <60 ml/min per 1.73 m^2^	eGFR 15 to <45 ml/min per 1.73 m^2^	eGFR ≥90 ml/min per 1.73 m^2^	eGFR 60 to <90 ml/min per 1.73 m^2^	eGFR 45 to <60 ml/min per 1.73 m^2^	eGFR 15 to <45 ml/min per 1.73 m^2^
Total cholesterol	−31.9 (−34.0 to −29.9)	−32.2 (−34.2 to −30.3)	−36.2 (−40.6 to −31.8)	−32.7 (−41.4 to −24.1)	−59.4 (−63.5 to −55.3)	−59.3 (−62.9 to −55.7)	−66.3 (−74.2 to −58.4)	−62.7 (−77.8 to −47.6)
ApoB	−40.5 (−42.8 to −38.3)	−42.5 (−44.7 to −40.3)	−44.9 (−49.9 to −39.9)	−42.1 (−51.1 to −33.0)	−39.7 (−42.0 to −37.4)	−40.1 (−42.3 to −38.0)	−42.3 (−47.0 to −37.7)	−40.3 (−48.8 to −31.7)
Non–HDL cholesterol	−45.1 (−47.8 to −42.3)	−46.8 (−49.4 to −44.2)	−49.8 (−55.8 to −43.8)	−47.3 (−58.4 to −36.2)	−61.8 (−65.9 to −57.8)	−62.4 (−65.9 to −58.9)	−66.4 (−74.0 to −58.9)	−65.7 (−80.7 to −50.7)
Triglycerides	−6.7 (−11.2 to −2.3)	−11.6 (−15.6 to −7.6)	−7.6 (−15.6 to 0.5)	−30.3 (−48.1 to −12.6)	−9.1 (−15.9 to −2.2)	−18.1 (−24.6 to −11.5)	−10.3 (−23.5 to 2.8)	−39.5 (−73.0 to −6.0)
Lp(a),^a^ nmol/L	−20.3 (−22.9 to −17.7)	−20.0 (−22.4 to −17.6)	−28.6 (−37.6 to −19.7)	−22.3 (−33.2 to −11.5)	−8.2 (−9.7 to −6.8)	−8.2 (−9.6 to −6.9)	−12.0 (−15.4 to −8.7)	−6.4 (−12.4 to −0.5)
Remnant cholesterol	−16.5 (−20.5 to −12.4)	−20.7 (−24.5 to −16.9)	−17.8 (−25.6 to −10.0)	−40.9 (−59.8 to −22.0)	−4.9 (−6.1 to −3.7)	−6.3 (−7.5 to −5.1)	−5.4 (−7.8 to −3.0)	−11.9 (−18.6 to −5.2)

Patients were classified into four categories based on their baseline eGFR. These categories align with Kidney Disease Improving Global Outcomes (KDIGO) GFR categories: ≥90 ml/min per 1.73 m^2^ (KDIGO GFR category G1); 60 to <90 ml/min per 1.73 m^2^ (KDIGO GFR category G2); 45 to <60 ml/min per 1.73 m^2^ (KDIGO GFR category G3A); and 15 to <45 ml/min per 1.73 m^2^ (KDIGO GFR category G3B–G4). None of the patients had GFR <15 ml/min per 1.73 m^2^, which aligns with KDIGO GFR category G5. ApoB, apolipoprotein B; CI, confidence interval; Lp(a), lipoprotein(a); LS, least squares.

a The percentage and absolute changes (least square mean [95% confidence interval]) in other atherogenic lipids except lipoprotein(a) from baseline to day 510 were analyzed using mixed-effects model for repeated measures without imputation, including fixed effects for treatment, visit, study, and treatment-by-visit interaction, and baseline value as a covariate. For lipoprotein(a), median (95% confidence interval) values at day 540 was provided from quantile regression models without imputation, with treatment and study as factors and baseline value as a covariate. All models were applied separately within each eGFR category. Treatment differences are presented for inclisiran − placebo.

#### Exploratory End Points

The proportions of patients experiencing reduction in eGFR by ≥30%, ≥40%, and ≥50% were not significantly different between inclisiran and placebo groups, regardless of eGFR category (*P* > 0.05 for all; Supplemental Table 2). The percentage change in eGFR over time showed no effect of inclisiran compared with placebo (Supplemental Figure 1).

### Safety

The safety population comprised 3655 patients classified by baseline eGFR. Inclisiran was well tolerated, with a safety profile similar to placebo, except for a higher proportion of patients with clinically relevant mild or moderate TEAEs at the injection site with inclisiran, regardless of baseline eGFR categories (Table 3 and Supplemental Tables 2 and 3). The most common TEAEs (≥5%) in both treatment arms were diabetes mellitus, nasopharyngitis, upper respiratory tract infection, and hypertension. TEAEs and treatment-emergent serious adverse events were more frequent in patients with low eGFR at baseline (Table 3). Frequent TEAEs with inclisiran were generally similar to placebo and consistent across the eGFR categories. Clinically relevant laboratory abnormalities were low and similar for inclisiran and placebo, independent of baseline eGFR category (Supplemental Table 3).

**Table 3:** Safety summary by baseline eGFR categories (safety population)

Parameter	eGFR ≥90 ml/min per 1.73 m^2^			eGFR 60 to <90 ml/min per 1.73 m^2^			eGFR 45 to <60 ml/min per 1.73 m^2^			eGFR 15 to <45 ml/min per 1.73 m^2^		
	Inclisiran (*n*=820), *n* (%)	Placebo (*n*=787), *n* (%)	RR (95% CI)	Inclisiran (*n*=793), *n* (%)	Placebo (*n*=814), *n* (%)	RR (95% CI)	Inclisiran (*n*=154), *n* (%)	Placebo (*n*=145), *n* (%)	RR (95% CI)	Inclisiran (*n*=66), *n* (%)	Placebo (*n*=76), *n* (%)	RR (95% CI)
Patients with ≥1 TEAE	630 (77)	574 (73)	1.1 (1.0 to 1.1)	614 (77)	647 (79)	1.0 (0.9 to 1.0)	131 (85)	127 (88)	1.0 (0.9 to 1.1)	55 (83)	61 (80)	1.0 (0.9 to 1.2)
Patients with ≥1 TESAE	146 (18)	152 (19)	0.9 (0.8 to 1.1)	159 (20)	193 (24)	0.8 (0.7 to 1.0)	46 (30)	40 (28)	1.1 (0.8 to 1.5)	23 (35)	34 (45)	0.8 (0.5 to 1.2)
Patients with ≥1 event leading to study discontinuation	18 (2)	14 (2)	1.2 (0.6 to 2.5)	24 (3)	11 (1)	2.2 (1.1 to 4.5)	2 (1)	6 (4)	0.3 (0.1 to 1.5)	1 (2)	4 (5)	0.3 (0.0 to 2.5)
**Patients with ≥1 clinically relevant TEAE at the injection site**	17 (2)	1 (0.1)	16.3 (2.2 to 122.3)	19 (2)	2 (0.2)	9.8 (2.3 to 41.7)	1 (0.6)	0	NA	1 (2)	1 (1)	1.2 (0.1 to 18.1)
Mild	11 (1)	1 (0.1)	10.6 (1.4 to 81.6)	12 (2)	1 (0.1)	12.3 (1.6 to 94.5)	0	0	NA	0	1 (1)	NA
Moderate	6 (0.7)	0	NA	7 (0.9)	1 (0.1)	7.2 (0.9 to 58.3)	1 (0.6)	0	NA	1 (2)	0	NA
Severe	0	0	NA	0	0	NA	0	0	NA	0	0	NA

Patients were classified into four categories based on their baseline eGFR ≥90 ml/min per 1.73 m^2^ (Kidney Disease Improving Global Outcomes [KDIGO] GFR category G1); 60 to <90 ml/min per 1.73 m^2^ (KDIGO GFR category G2); 45 to <60 ml/min per 1.73 m^2^ (KDIGO GFR category G3A); and 15 to <45 ml/min per 1.73 m^2^ (KDIGO GFR category G3B–G4). None of the patients had GFR <15 ml/min per 1.73 m^2^, which aligns with KDIGO GFR category G5. Risk ratio of inclisiran/placebo within each baseline eGFR subgroup. CI, confidence interval; NA, not available; RR, risk ratio; TEAE, treatment-emergent adverse event; TESAE, treatment-emergent serious adverse event.

## Discussion

This *post hoc* pooled analysis of 3660 patients from the pivotal phase 3 ORION-9, ORION-10, and ORION-11 trials demonstrated that inclisiran, compared with placebo, reduced LDL cholesterol significantly and consistently with a favorable safety profile, across categories of kidney function spanning CKD stages 1-4. Inclisiran, administered twice yearly (after initial and 3-month doses) for up to 18 months provided sustained and effective LDL cholesterol reductions after the second dose until the end of the study (placebo-corrected time-adjusted percentage change) of 48.4%, 51.8%, 55.6%, and 50.4% among patients with eGFR ≥90, 60 to <90, 45 to <60, and 15 to <45 ml/min per 1.73 m^2^, respectively. These findings align with the overall pooled population results, where the time-adjusted percentage reduction in LDL cholesterol was 50.5%.^16^

Compared with individuals with normal kidney function, patients with CKD typically exhibit distinct differences in their lipid profiles, including elevated triglycerides, increased levels of Lp(a), and reduced HDL cholesterol.^19–21^ In individuals with impaired kidney function, triglycerides, Lp(a), and non–HDL cholesterol may correlate with cardiovascular risk.^4,22^ Our analysis showed significant reductions in each of these atherogenic lipoproteins with inclisiran across all eGFR categories.

Non–HDL cholesterol, a comprehensive measure of cholesterol content in ApoB-containing particles, was reduced by >45% with inclisiran. ApoB, which represents the total number of atherogenic particles, was reduced by >42% with inclisiran versus placebo. Moreover, remnant cholesterol, which is a proatherogenic risk factor in individuals with CKD,^8^ was reduced by >16% (up to >40% in eGFR 15 to <45 ml/min per 1.73 m^2^ group) with inclisiran versus placebo. These changes are important in a population where cardiovascular risk reduction can be challenging.

Assignment to inclisiran versus placebo did not affect the trajectory of kidney function, with no difference in the numbers of patients with reductions in eGFR ≥30%, ≥40%, or ≥50% (Supplemental Table 4).

A prior analysis showed transient greater plasma inclisiran concentration in patients with kidney dysfunction versus patients with normal kidney function,^17^ but in the current analysis, CKD severity did not affect the reduction of LDL cholesterol with inclisiran. A meta-analysis found that high-intensity statins significantly reduced the decline in eGFR compared with placebo, usual care or no-statin treatment. However, this effect was not observed with moderate-intensity or low-intensity statins.^23^ Nonetheless, the KDIGO guidelines endorse moderate-intensity or high-intensity statin in patients with CKD.^6^ Analyses of the Further Cardiovascular Outcomes Research With PCSK9 Inhibition in Subjects With Elevated Risk and ODYSSEY OUTCOMES trials corroborate a lower usage of high-intensity statins in populations with advanced CKD,^10,11^ which aligns with the present study patients (Table 1 and Supplemental Table 5). Anti-PCSK9 monoclonal antibodies have shown effective LDL cholesterol reduction in combination with statin in hypercholesterolemia patients, regardless of impaired kidney function,^4,10^ similar to inclisiran results in patients with CKD.

Inclisiran was generally well tolerated regardless of CKD severity, with similar proportions of TEAEs and treatment-emergent serious adverse events reported between treatment arms and across eGFR subgroups. The safety profile was consistent with the overall pooled population.^16^ TEAEs at the injection site were more common with inclisiran but were mild to moderate, with none being severe. A pooled analysis of seven clinical trials with 3576 patients showed that long-term inclisiran treatment for up to 6 years was well tolerated without new safety findings.^24^ Furthermore, ORION-8 demonstrated a favorable long-term safety and tolerability profile with up to 6.8 years of inclisiran exposure.^25^

There are limitations to this pooled analysis. There were very few patients with severe form of the disease, with only 142 patients having a baseline eGFR 15 to <45 ml/min per 1.73 m^2^. Furthermore, there were only 14 patients with eGFR of 15 to <30 ml/min per 1.73 m^2^, and none had an eGFR <15 ml/min per 1.73 m^2^. Hence, estimates of efficacy, safety, and tolerability of inclisiran are imprecise in CKD stages 3B and 4 and unknown in CKD stage 5. Furthermore, data on urinary albumin-to-creatinine ratio or urinary protein-to-creatinine ratio were not collected in the original ORION-9, ORION-10, and ORION-11 studies and are therefore unavailable for this *post hoc* analysis. This limits our ability to evaluate kidney damage markers and their relationship to LDL cholesterol lowering. To ensure accurate kidney function classification despite this limitation, eGFR was calculated using the 2021 CKD Epidemiology Collaboration equation. In addition, cardiovascular outcome data were not captured in these trials, which restricts conclusions regarding long-term cardiorenal benefit. Ongoing placebo-controlled cardiovascular outcomes trials, including ORION-4,^26^ VICTORION-1 Prevent,^27^ and VICTORION-2 Prevent,^28^ will provide further insights into efficacy, safety, and tolerability of inclisiran in high-risk populations.

Inclisiran, administered twice-yearly (after initial and 3-month doses) with maximum-tolerated statins or other oral lipid-lowering therapies, provides sustained and effective LDL cholesterol lowering with favorable safety for a broad range of patients with atherosclerotic cardiovascular disease, including those with advanced CKD. However, given the limited sample size in advanced CKD stages, these findings should be interpreted with caution.

## Data Availability

Data Type: Clinical Trial Data. Reason for Restricted Access: Requests for patient-level data and supporting clinical documents will be considered by an independent review panel based on scientific merit. All data provided are anonymized to respect the privacy of patients who have participated in the trial in line with applicable laws and regulations. This availability of these trial data is according to the criteria and process described on www.clinicalstudydatarequest.com.

## Declarative Statements

This study includes clinical experimentation and received Institutional Review Board or Ethics Committee approval. All patients provided written informed consent. This study includes clinical experimentation and complies with the Declaration of Helsinki.

## Supplemental Material

Supplemental Summary 1. List of investigators and study sites.

Supplemental Appendix 1. Details of multiple imputation methods and missing data.

Supplemental Table 1. Treatment-by-eGFR subgroup interaction effects (ITT population).

Supplemental Table 2. Frequent TEAEs (≥5% in any treatment group) by baseline eGFR (safety population).

Supplemental Table 3. Clinically relevant laboratory measurements by baseline eGFR (safety population).

Supplemental Table 4. Proportion of patients achieving exploratory eGFR thresholds at any visit by baseline eGFR (ITT population).

Supplemental Table 5. Statin use at baseline with dose by baseline eGFR (ITT population).

Supplemental Figure 1. Percent change in eGFR from baseline (ITT population).
